# A Community Outbreak of Cryptosporidiosis in Sydney Associated with a Public Swimming Facility: A Case-Control Study

**DOI:** 10.1155/2011/341065

**Published:** 2011-12-13

**Authors:** Darren J. Mayne, Kelly-Anne Ressler, Diane Smith, Gareth Hockey, Susan J. Botham, Mark J. Ferson

**Affiliations:** ^1^South Eastern Sydney Illawarra Public Health Unit, Health Reform Transition Organisation—Southern, Locked Mail Bag 9, Wollongong, NSW 2526, Australia; ^2^South Eastern Sydney Illawarra Public Health Unit, Health Reform Transition Organisation—Southern, Locked Bag 88, Randwick, NSW 2031, Australia; ^3^School of Public Health and Community Medicine, Faculty of Medicine, University of New South Wales, Level 2 and 3 Samuels Building, UNSW Sydney, 2052 NSW, Australia; ^4^School of Medicine Sydney, University of Notre Dame Australia, 160 Oxford Street, Darlinghurst NSW 2010, Australia

## Abstract

In February, 2008, the South Eastern Sydney Illawarra Public Health Unit investigated an outbreak of cryptosporidiosis within the south east region of Sydney, Australia. Thirty-one cases with laboratory-confirmed cryptosporidiosis and 97 age- and geographically matched controls selected by random digit dialling were recruited into a case-control study and interviewed for infection risk factors. Cryptosporidiosis was associated with swimming at Facility A (matched odds ratio = 19.4, 95% confidence interval: 3.7–100.8) and exposure to household contacts with diarrhoea (matched odds ratio = 7.7, 95% confidence interval: 1.9–31.4) in multivariable conditional logistic regression models. A protective effect for any animal contact was also found (matched odds ratio = 0.2, 95% confidence interval: 0.1–0.7). *Cryptosporidium hominis* subtype IbA10G2 was identified in 8 of 11 diagnostic stool samples available for cases. This investigation reaffirms the importance of public swimming pools as potential sources of *Cryptosporidium* infection and ensuring their compliance with water-quality guidelines. The protective effect of animal contact may be suggestive of past exposure leading to immunity.

## 1. Introduction

*Cryptosporidium* is a protozoan parasite that causes human cryptosporidiosis [1] and is a major source of acute gastrointestinal disease outbreaks associated with recreational water use in Australia [2–4] and internationally [5–8]. Cryptosporidiosis typically presents as profuse and watery diarrhea, but asymptomatic infection is common and a source of infection for others [1]. *Cryptosporidium* oocysts are well adapted to waterborne transmission by the faecal-oral route in recreational water use settings. *Cryptosporidium* oocysts have a low infective dose of between 10 and 30 in healthy adults [9, 10], are highly concentrated in human stool and may be excreted for weeks after symptoms resolve [11, 12], and are resistant to halogen disinfection [13] at free chlorine levels that are recommended for treating recreational water sources (1–3 mg/L) [14].

In late January, 2008, public health unit staff identified an increase in laboratory notifications for cryptosporidiosis amongst residents in Sydney's eastern and southern local government areas of Botany, Randwick, Sydney, Waverley, Woollahra, Hurstville, Kogarah, and Rockdale (referred to as south eastern Sydney). Many of the cases reported potential exposure at the same public swimming facility (Facility A) during the twelve days prior to disease onset in standard public health follow-up interviews.

An inspection of Facility A's management procedures, operating logs and five pools in February, 2008 by environmental health officers indicated that chlorine levels were frequently below the recommended level (1–3 mg/L) in the New South Wales *Public Swimming Pool and Spa Pool Guidelines *[14] for two indoor pools during the period of increased cryptosporidiosis notifications. A water sample taken for bacteriological testing from an indoor heated pool during the inspection also tested positive for *Pseudomonas aeruginosa*. The operator complied with public health unit recommendations to voluntarily close the pool, which was drained, sanitised, refilled and superchlorinated overnight to a level of 10 mg/L. The public health unit also requested that the operator engage a qualified pool consultant to assess the adequacy of their plant and equipment with regards to turnover rates, filtration, disinfection, bather loads, and management practices. Ongoing monitoring of the water quality in Facility A's pools was initiated, and retesting in early March indicated all pools were free of indicator bacteriological contamination. Notifications of cryptosporidiosis continued to rise throughout February, 2008 in Sydney's eastern and southern suburbs, and so, a formal investigation of the aetiology and risk factors of the outbreak was initiated.

## 2. Materials and Methods

A case-control study was undertaken using laboratory notifications made to the South Eastern Sydney Illawarra Public Health Unit. A case was defined as any person living in south eastern Sydney with laboratory confirmed cryptosporidiosis diagnosed by microscopy or antigen detection on the basis of stool specimens collected between 1 February and 31 March, 2008. Cases prior to February, 2008 were excluded due to the likelihood of recall bias resulting from delay between disease onset and study initiation in early March, 2008. This also ensured that statistical tests were independent of the data used to generate study hypotheses from routine public health follow-up with these early cases. Case selection was not restricted by clinical symptoms to ensure consistency with state and national case definitions [15, 16].

Random digit dialling was used to match three controls from the study population to each case. Controls were matched to cases within the age groups ≤5, 6–24, and ≥25 years; controls had to be within 5 years of the matched case for ages 6–24 years and within 10 years for cases aged ≥25 years. Controls also had to live in the same or an adjacent suburb to their matched case and deny gastrointestinal symptoms during the 12 days preceding disease onset for their matched case.

A list of random telephone numbers for the study area was created by permutating the last four digits of telephone numbers from case suburbs. The list was randomly ordered and split into subsets of 1,000 numbers, which were dialled sequentially by public health unit staff: a new list was started only when the current list had been exhausted. Up to three calls were made to each number to identify households with an eligible control. Only one control was recruited per household. The person with the closest age and suburb match to a study case was selected when households contained multiple eligible controls.

Cases and controls were interviewed by telephone using a standardised questionnaire that was based on the New South Wales Health Department's cryptosporidiosis case investigation form [17]. The interview collected demographic data, clinical information for cases, and risk factors for infection. Risk factors included exposure to household contacts with diarrhoea and vomiting, sources of drinking water, consumption of untreated water, swimming in public and private pools and open bodies of water (e.g., creeks and dams), contact with animals, farm visitations, and domestic and international travel. Exposure data were collected for the 12 days prior to disease onset for cases and their matched controls.

Most case interviews occurred within seven days of notification to the public health unit, and control interviews were completed as soon as an eligible individual was identified by random digit dialling. Interviews with children under 18 years of age were conducted by proxy through a parent or guardian. Cases and controls were informed that the study was investigating possible reasons for increased diarrhoeal disease in south eastern Sydney in 2008 but were not informed of specific study hypotheses. It was not possible to blind interviewers to either the study hypotheses or the case status of interviewees.

Associations between cryptosporidiosis and risk factors for infection were assessed using matched odds ratios (MORs) estimated from conditional logistic regression models [18]. It was not possible to simultaneously adjust for all risk factors in a multivariate model as sparse data and moderate correlations between exposure variables resulted in numerically unstable solutions. We therefore used forward selection to arrive at a final multivariable model containing all exposure terms significantly associated with *Cryptosporidium* infection after conditioning on variables previously selected into the model [19]. The statistical significance of all models was assessed using the likelihood ratio test (*χ*_LR_^2^) and an alpha level of 0.05.

In addition to the epidemiological investigation, residual stool samples were obtained for 11 of 31 study cases from diagnosing pathology laboratories. These were sent to the Department of Biological Sciences at Macquarie University, Australia, for species identification using terminal restriction fragment length polymorphism [20] and subtyping using nested polymerase chain reaction and sequencing of the glycoprotein 60 gene [21].

Ethical review was not required for this investigation, as it was authorised under the New South Wales *Public Health Act 1991*.

## 3. Results

Between 1 February, 2008 and 31 March, 2008, there were 43 laboratory notifications of cryptosporidiosis within the study area. The annualised notification rate for the study period was 51 per 100,000 (95% CI: 32.9 to 75.2), which was 2.9 times higher than the mean notification rate of 17.4 per 100,000 for the same period from 2003 to 2007 (95% CI: 12.5 to 23.5; *χ*_LR_^2^ = 6.33, DF = 1, *P* < 0.0001). The median age of notified cases was 5.2 years (range: 0.4 to 90.1 years), and 55% were males. Forty-six per cent of notifications were among children aged less than five years.

Thirty-one (72%) of the 43 notifications were included in the study: eight (19%) could not be contacted, while four (9%) had been overseas for the entire exposure period and were excluded. Nineteen of the 31 cases (61%) were diagnosed by microscopy and antigen detection, five (16%) by microscopy only, and seven (23%) by antigen detection only. One family cluster of three cases was included in the study, as all family members had risk factors for infection in addition to household contact, including swimming at public pools and beaches. The median age of study cases was 4 years (range 0.7 to 65 years), with 39% aged less than five years and 55% male (55%). Participant demographics did not differ significantly to nonparticipant characteristics.

One hundred and seven controls were recruited from 508 households contacted, giving an overall response rate of 21.1%. The response rate for eligible individuals within contacted households could not be estimated, because a refusal to participate was mostly initiated at the household level; that is, before inclusion-criteria data could be collected. Three controls were subsequently excluded due to incorrect age matching (*n* = 2) or the presence of gastrointestinal symptoms during their matched case's exposure period (*n* = 1); seven controls matched to cases that could not be contacted for interview were also excluded. Three controls were matched to 28 cases, 4 to 2 cases and 5 to 1 case. Controls comprised a lower proportion of males (47%, *n* = 46) compared to cases (55%, *n* = 17) and were slightly older (median = 5 years, range 0.3 to 69 years) than cases (median = 4 years, range = 0.7 to 65 years). Forty-four (45%) controls were recruited from the same suburb as their matched case.

Thirty of the 31 cases (97%) reported diarrhoeal symptoms, and 9 (29%) were still ill at the time of interview; the one case without diarrhoea had other gastrointestinal symptoms that prompted testing. The median length of illness for people whose symptoms had resolved was 14 days (range: 2 to 30 days). Four children aged 0–5 years were admitted to hospital for lengths of stay ranging from 1 to 6 days (median 1.5 days). Matched odds ratios and 95% confidence intervals for risk factors associated with *Cryptosporidium* infection are shown in Table 1. In unadjusted analyses infection was significantly associated with contact with a household member experiencing diarrhoea (42% versus 13%; *χ*_LR_^2^ = 11.5, degrees of freedom (DF) = 1, *P* = 0.0007) or vomiting (29% versus 7%; *χ*_LR_^2^ = 9.3, DF = 1, *P* = 0.002), any swimming (84% versus 58%; *χ*_LR_^2^ = 7.9, DF = 1, *P* = 0.005), and swimming at Facility A (48% versus 11%; *χ*_LR_^2^ = 21.3, DF = 1, *P* < 0.0001). Infection was not associated with swimming at a facility other than Facility A (23% versus 22%; *χ*_LR_^2^ = 0.02, DF = 1, *P* = 0.90) or in a natural water body (35% versus 35%; *χ*_LR_^2^ = 0.0001, DF = 1, *P* = 0.99). Any contact with animals was found to be protective against *Cryptosporidium* infection (32% versus 63%; *χ*_LR_^2^ = 8.9, DF = 1, *P* = 0.003).

A final multivariable model was arrived at using the forward selection method and included swimming at Facility A (*χ*_LR_^2^ = 19.5, DF = 1, *P* < 0.0001), contact with a household member with diarrhoea (*χ*_LR_^2^ = 10.1, DF = 1, *P* < 0.002), and contact with any animals (*χ*_LR_^2^ = 7.5, DF = 1, *P* < 0.006). Matched odds ratios and 95% confidence intervals from this final model are shown in Table 1. Swimming at Facility A and contact with a household member with diarrhoea were associated with increased risk of cryptosporidiosis, while contact with any domestic animals was protective against infection. There was no evidence of effect modification in this final model.

*Cryptosporidium* oocysts were detected in 8 of the 11 residual diagnostic stool samples from cases sent for further molecular analysis. These eight isolates were all identified as *Cryptosporidium hominis* subtype IbA10G2. Two of the three samples in which oocysts could not be detected were diagnosed using antigen detection and microscopy, and the third was diagnosed by antigen detection only.

## 4. Discussion

Notification of cryptosporidiosis in south eastern Sydney residents from February to March, 2008 was strongly associated with swimming at Facility A and person-to-person transmission in the household environment. The outcomes of this study are consistent with previous epidemiological and environmental outbreak investigations in New South Wales that have linked swimming in public pools to higher cryptosporidiosis risk [2–4, 22], and highlight the importance of ensuring operator compliance with water-quality guidelines and risk minimisation protocols through awareness raising and active enforcement, especially during seasonal peak periods when bather loads are high [14, 23]. Operators should be encouraged to promote self-exclusion amongst patrons with diarrhoea and in the two weeks following resolution of symptoms by displaying appropriate signage at entry points and within the facility. This signage should also reinforce the importance of non-toilet-trained infants wearing waterproof pants over swimmers, as well as discouraging people with faecal incontinence from swimming [23].

The strong association between cryptosporidiosis and person-to-person contact, particularly within households, has also been observed for sporadic cases of cryptosporidiosis in Australia [24]. This study reinforces the importance of delivering and reinforcing effective personal prevention measures such as adequate hand washing in reducing household spread. This is especially relevant to households with young children. People must also be encouraged to refrain from using recreational water facilities—including pools, beaches, dams, and other water bodies—while ill and for the two weeks following resolution of diarrhoeal symptoms.

The protective effective of any animal contact in this study has also been reported for sporadic cryptosporidiosis cases in southern Australia and may be suggestive of past exposure leading to immunity [24]. Preliminary support for this hypothesis comes from a 2005 study showing populations with moderately strong serological responses to 15/17- and 27-kDa *Cryptosporidium* antigen groups were at lower risk of endemic gastrointestinal illness [25]. However, this evidence is still largely suggestive, and further research is required in this area.

Molecular analysis indicated that *Cryptosporidium hominis* subtype IbA10G2 was the likely causative agent of this outbreak, as it was identified in all residual diagnostic stool samples from confirmed cases in which oocysts were detectable. Molecular and epidemiological analysis have also implicated this subtype in a cryptosporidiosis outbreak in the United States associated with exposure to a community swimming pool [5]. Subtype IbA10G2 is widely distributed throughout Australia [26] and is a common cause of cryptosporidiosis in New South Wales [21, 27], which may reduce its discriminatory value in outbreaks when corroborating environmental evidence is limited. Despite this constraint, the molecular evidence reported here supports the internal consistency of the epidemiological findings.

Oocysts could not be recovered from 3 of the 11 diagnostic stool samples that had previously tested positive for *Cryptosporidium* infection by either antigen detection only (*n* = 1) or antigen detection and microscopy (*n* = 2). False-positive results can occur with antigen immunoassays for *Cryptosporidium* [28–30], which may explain the inconsistency between the diagnostic and molecular findings for the case tested using antigen detection only. However, antigen reactions confirmed by microscopy have reported specificities of 100% for *Cryptosporidium hominis* [28], making it unlikely that the two cases diagnosed using both these methods are false positives. It is feasible that oocysts were insufficiently concentrated in these samples to be purified by the typing laboratory's modified sucrose floatation extraction method. Comparative studies of extraction methods using *Cryptosporidium parvum* oocysts have demonstrated that the sucrose floatation method is less efficient than sodium chloride and water-ether floatation methods, especially at low oocysts concentrations [31, 32]. This warrants further investigations given its potential impact for case classification and exclusion in outbreak investigations.

There are a number of limitations to this study. First, public health officers who interviewed cases and controls were not blinded to the purpose of the study; thus, it is possible that staff may have been more diligent at eliciting Facility A swimming exposures for cases than controls. Second, the delay between case disease onset and interview was, on average, longer for controls than cases: this delay may have induced recall bias in matched controls, which are also likely to be less motivated to recall exposures than cases [19]. Third, the overall response rate for controls to participate in the study was low (21%), which may affect their representativeness of the study base from which the cases arose. Fourth, this study only used laboratory-confirmed cryptosporidiosis cases and explicitly excluded controls reporting diarrhoea during the exposure period; as such, it was not possible to estimate the magnitude of the outbreak in the study population. Fifth, laboratory testing for cryptosporidiosis was limited to cases, and so, there is potential for individuals with asymptomatic infection during the period for which risk factors were elicited to be included as controls.

We believe the observed association between cryptosporidiosis and swimming at Facility A is unlikely to be an artefact of the first two potential sources of bias, as (a) the importance of eliciting all cryptosporidiosis exposures was stressed during interviewer training and (b) cryptosporidiosis risk remained significantly higher for cases in sensitivity analyses assuming 10%–15% misclassification of exposure to Facility A in control participants. Likewise, inclusion of controls with asymptomatic infection would only be expected to bias this association away from the null if misclassification were higher for nonexposed individuals [33], but there is no reason to suspect differential misclassification in the current study. Our low response rate is consistent with reports of increasing difficulty in recruiting controls and decreasing participation rates in epidemiological studies [34, 35], especially those using population controls [36]. There is a risk of selection bias and ascertainment bias due to the low response rate and nonverification of control status, respectively, especially if participation is related to swimming exposure. However, the effect size, consistency of results with previous research [2, 3, 22, 24], converging epidemiological and microbiological evidence, and the identification of water-quality failure and water treatment problems suggest that the observed association for swimming at Facility A is unlikely to be attributable to bias.

## 5. Conclusion

This outbreak highlights the ongoing need for local health authorities to ensure appropriate disinfection processes and control measures are implemented by pool operators by promoting adherence to relevant water-quality guidelines and protocols for the operation of public swimming pools. There is also a need to promote infection control practices in the general community to ensure that persons with diarrhoeal illness do not swim whilst unwell and for two weeks after diarrhoea has ceased. The importance of thorough hand washing to prevent person-to-person transmission in households also needs to be emphasised in the general community. In response to the consultant's report recommended by the public health unit, Facility A upgraded its water filtration systems, reconstructed one outdoor pool and refurbished another, and installed a free chlorine chemical controller system. No new epidemic cases of cryptosporidiosis have been linked to this facility since the implementation of these measures despite a large community-wide cryptosporidiosis outbreak in the Sydney metropolitan area in 2009.

## Figures and Tables

**Table 1:** Unadjusted and adjusted matched odds ratios for risk factors associated with *Cryptosporidium* infection.

	Cases (Total = 31)		Controls (Total = 97)		Matched odds ratio			
	Exposed	Percent	Exposed	Percent	Unadjusted	95% CI	Adjusted*	95% CI
Female	14	45	51	53	0.8	0.4–1.7		
Contact with people with diarrhoea	13	42	13	13	5.6	1.9–16.0	7.7	1.9–31.4
Contact with people with vomiting	9	29	7	7	5.9	1.8–19.3		
Attended childcare or preschool	12	39	27	28	1.7	0.7–4.5		
Drank mains water	31	100	94	97	Undefined			
Drank bottled water	1	3	10	10	0.2	0.0–2.0		
Drank untreated water	0	0	0	0	Undefined			
Any swimming	26	84	56	58	4.1	1.4–12.6		
Swam in pool at Facility A^†^	15	48	11	11	15.8	3.5–70.9	19.4	3.7–100.8
Swam in pool other than at Facility A	7	23	21	22	1.1	0.4–3.0		
Swam in other water body (e.g., dam and ocean)	11	35	34	35	1.0	0.4–2.3		
Animal contact	10	32	61	63	0.3	0.1–0.7	0.2	0.1–0.7
Visited a farm	0	0	9	9	Undefined			
International travel	0	0	3	3	Undefined			
Domestic travel	0	0	23	24	Undefined			

^∗*︢*^Adjusted for all other variables in the model; ^†^No other pools were associated with diagnosis of cryptosporidiosis disease; CI = confidence interval.
